# Assessing toxic leadership in high-performance sports: Psychometric validation of a Swedish Version of the Toxic Leadership Scale for Sports (TLS-S)

**DOI:** 10.1371/journal.pone.0343533

**Published:** 2026-03-06

**Authors:** Carolina Lundqvist, Saga Johansson

**Affiliations:** 1 Department of Behavioural Sciences and Learning, Linköping University, Linköping, Sweden; 2 Athletics Research Center, Linköping University, Linköping, Sweden; Mugla Sitki Kocman University: Mugla Sitki Kocman Universitesi, TÜRKIYE

## Abstract

This study evaluated the internal consistency and factorial validity of a sport-adjusted 15-item version of the Toxic Leadership Scale (TLS-S). The sample consisted of 189 Swedish elite team sport athletes (56 females, 132 males, 1 preferred not to disclose; mean age = 17.9 years, SD = 3.3) from 21 different teams (ice hockey n = 68; soccer n = 60; American football n = 26; basketball n = 15; handball n = 14; volleyball n = 3; other team sports not specified or multiple sports n = 3). Participants competed at different performance levels: sub-elite (n = 15), national junior elite (n = 105), international junior elite (n = 26), national senior elite (n = 30), international senior elite (n = 12), and other not specified (n = 1). Results showed adequate internal consistency (ω_t_ > .70) for the total TLS-S score and all subscales except the authoritarian leadership subscale (ω_t_ = .61). Confirmatory factor analysis indicated acceptable model fit for both a five-factor and a one-factor model. While the five-factor model (χ²(80)=135.87, p < .001; CFI = .98; RMSEA = .062 [90% CI: .044–.080]; SRMR = .044) was superior to the one-factor solution (difference test: χ²(10)=53.60, p < .001), intercorrelations across subscales were high (range: .73−.92). A bifactor model supported a unidimensional structure (explained common variance = .79; percentage of uncontaminated correlations = .86). The findings provide psychometric evidence for the TLS-S and support a unidimensional structure. This study constitutes an important initial step toward facilitating the quantitative assessment of toxic leadership within sport environments, with the TLS-S emerging as a promising instrument for this purpose.

## 1. Introduction

In high-performance sport, athletic success depends not only on physical capacity, talent, and technical skills but also on the quality of leadership. Effective leadership can enhance team cohesion, foster personal development, and support sustainable performance [1,2]. However, when cultural norms within high-performance sport endorse the belief that strict, dominant, and controlling leadership is essential for sports success, destructive and toxic leadership may become normalized or even incentivized by sports organizations [3–6]. Such practices can contribute to dysfunctional team dynamics, fear-based relationships between leaders and followers, adverse health outcomes (e.g., psychological distress, injuries, diminished self-esteem), early career termination or turnover as well as compromised performance and negative organizational consequences [3,7–9]. While traditional leadership frameworks (e.g., situational leadership, the social identity approach, transformational leadership theory) have been applied in sport to explain how coaching influences athlete and team functioning,[e.g., 10–12] these approaches primarily emphasize leadership effectiveness in promoting wellbeing and performance. In contrast, the more destructive and toxic dimensions of leadership, those that undermine athlete welfare, team cohesion, and performance, have only recently begun to receive systematic attention in sport research [3]. Consequently, the destructive and toxic side of sports leadership remains comparatively underexplored and measurement tools are lacking. To advance a comprehensive understanding of leadership in sport, researchers must address both its constructive and destructive dimensions, acknowledging the dual potential of leadership to either support or undermine athlete welfare, health, and short- and long-term performance.

### 1.1. Definitions of toxic leadership

Toxic leadership is well documented in domains outside the sports sector (e.g., academia, business, politics, the military) that share historical connections or organizational similarities with sports, such as a performance-oriented nature and a hierarchical power structure [8,13–17]. Although there is currently no universally accepted definition of toxic leadership, and numerous partially overlapping constructs related to destructive leadership exist (e.g., abusive supervision, despotic leadership, tyrannical leadership, leader narcissism, derailed leadership), these conceptualizations converge on a core premise: leaders are considered to engage in, or intend to engage in, behaviors perceived as harmful or destructive to one or more followers or the organization [8,9,18]. The term toxic leadership typically denotes leadership considered ‘poisonous’ to those within its sphere of influence and is conceptualized as a distinct subset within the broader framework of destructive leadership [18,19].

Toxic leadership is associated with self-serving, authoritarian, manipulative, narcissistic, ignorant, and exploitative behaviors, whereby leaders abuse power for personal gain, prioritizing their own interests and goals over collective needs [7–9,14,16,17,20]. Lipman-Blumen [20] defined toxic leaders as ‘individuals, who by dint of their destructive behaviors and dysfunctional personal qualities generate a serious and enduring poisonous effect on the individuals, families, organizations, communities, and even societies they lead’ (p. 30). Similarly, Gandolfi and Stone [7] characterize toxic leadership as an ‘intentional or unintentional series of acts that undermine and discourage those followers who genuinely seek to carry out the mission and vision of the organization, who then become stifled in the process of achievement by self-serving leaders who put missional or personal gain above the needs of followers, creating a demoralized state that deteriorates organizations from the inside out’ (p. 25). Building on definitions from non-sports contexts and recognizing through a scoping review that research on destructive and toxic leadership in sports remains at a nascent stage of development, Lundqvist et al. [3] introduced toxic leadership in high-performance sports as ‘a broad umbrella term encompassing various destructive, harmful, or socially undesirable leadership characteristics, behaviors, or intentions, as well as aligned processes that enable and maintain such leadership within the sports or organizational context, ultimately resulting in perceived or actual short- or long-term detrimental outcomes for affected individuals, organizations, or communities’ (p. 21).

Toxic leadership is not a single behavior or personality trait, and toxic leaders need not exhibit destructive behaviors in all situations, nor employ the same destructive behaviors consistently [14,16,20,21]. Rather, it is argued to represent the cumulative effect of demotivating actions that erode morale and create an unhealthy and unproductive organizational climate over time [14]. A one‑sided focus on individual characteristics or traits addresses the symptoms of toxicity, but not its underlying systemic causes and how organizational culture and climate can foster toxic leadership and shape subsequent behaviors and outcomes [17,22]. Thoroughgood et al. [19] criticized leader-centric approaches and argued for integrative views on leadership phenomena that involve interactions between flawed, toxic, or ineffective leaders, susceptible followers, and conducive environments [19,23]. Destructive leadership is typically characterized by individual-level behaviors that negatively target followers or subordinates, whereas toxic leadership emphasizes organizational-level dysfunction and its systemic consequences [19,21,23]. Thus, toxic leadership links harmful leader behaviors to their broader impact on followers, organizations, and the surrounding environment that collectively enable and perpetuate toxicity within organizational systems [23]. This dynamic shapes organizational culture, affects subordinates’ experiences, and impacts both organizational and individual performance outcomes [17].

### 1.2. Assessment of toxic leadership in high-performance sports

A limiting factor in advancing empirical understanding on leaders perceived as toxic in high-performance sport settings, is the current lack of validated measurement tools [3]. Leadership scales traditionally used in sports research, such as the Leadership Scale for Sports [24], the Coaching Behaviour Scale for Sport [25], and the Differentiated Transformational Leadership Inventory [26], generally do not include items that explicitly assess toxic leadership dimensions. To date, much of the available literature on various destructive leadership practices in high-performance sports has relied on qualitative methodologies, which, while valuable for capturing nuanced experiences, may limit the ability to quantify prevalence and expand the theoretical understanding of toxic leadership and its impact (e.g., health, performance, athlete dropout/staff turnover) on individuals, teams and organizations in high-performance sports settings [3].

The Toxic Leadership Scale (TLS) was specifically developed to assess toxic leadership, originally for military and civilian contexts such as business [27,28], and has yet not been utilized within sports. The TLS is a multidimensional measure intended to capture followers’ perceptions of core dimensions of toxic leadership, based on the assumption that toxic leadership comprises a constellation of behaviors that predictably undermine subordinate groups. The scale is grounded in the operationalization of toxic leaders as ‘narcissistic, self-promoters who engage in an unpredictable pattern of abusive and authoritarian supervision’ (p. 57) [27]. Accordingly, the TLS incorporates elements of self-promotion, unpredictability, abusive supervision, narcissism, and authoritarianism. An underlying assumption is that the destructive behaviors of toxic leaders extend beyond direct targets; by shaping the organizational climate, these behaviors may negatively affect a broader group of individuals, including those who merely witness such conduct [27]. In light of qualitative descriptions of toxic leadership noted in the nascent yet evolving field of research on toxic leadership in the sports domain [3], exploring the psychometric properties of the TLS adapted for use in a sports context could not only enable quantitative assessment of toxic leadership dimensions in sports research but also support the development of more effective strategies for identifying and mitigating harmful leadership practices in athletic environments. The purpose of this study was therefore to conduct an initial psychometric evaluation of the internal consistency and factorial validity of the TLS when adapted for use in sports settings (TLS-S). Since toxic leadership is closely related to culture and the climate in group settings, we chose to focus on team sports in this study to ensure that the athletes responding to the scale were embedded in a team context where leadership can exert influence on the group climate.

## 2. Materials and methods

This study employed a cross-sectional design, using a web-based survey administered to team sport athletes playing at the national or international elite level within their respective age categories.

### 2.1. Participants

To be eligible for inclusion in this study, participants were required to meet the following criteria: (a) be at least 15 years of age; (b) compete at a national or international elite level in a team sport, as determined by league classification; and (c) possess sufficient proficiency in Swedish to comprehend the survey content. Participants were excluded from the study if they reported experiencing severe mental health issues requiring active psychiatric treatment (e.g., substance abuse, psychotic disorders, obsessive-compulsive disorder or similar conditions) or if they were undergoing a significant personal crisis (e.g., divorce, death within the family or another close relationship, or similar serious events) at the time of data collection. Eligibility was assessed through initial screening questions. A total of 202 Swedish team sport athletes completed the survey, of whom 13 reported yes on one exclusion criterion (severe mental health issue, n = 10; personal crisis, n = 3) and were excluded from this study. After exclusion of non-eligible participants, the study sample consisted of 189 team sport athletes (females: n = 56; males: n = 132; prefer not to disclose: n = 1; mean age: 17.9 years, SD = 3.3).

The study sample represented athletes from 21 different teams in various team sports: ice hockey (n = 68), soccer (n = 60), American football (n = 26), basketball (n = 15), handball (n = 14), volleyball (n = 3), and other team sports not specified or multiple sports (n = 3). Participants reported competing at different performance levels: sub-elite (n = 15), national junior elite (n = 105), international junior elite (n = 26), national senior elite (n = 30), international senior elite (n = 12), and other not specified (n = 1). On average, athletes reported training 11.3 (SD = 5.5) hours per week and participating in 3.9 (SD = 2.9) games per week. The average number of coaches associated with each team was 4.2 (SD = 1.4), with most athletes indicating that their head coach was male (n = 175; 92.6%).

### 2.2. Measures

Demographics collected were self-reported sex, age, team sport practiced at an elite level, performance level, average/training hours in their main sport/week, average games played/week, number of coaches associated to the players main team, sex of the head coach for the players main team.

The Toxic Leadership Scale [27,28] is available in two formats: a full version comprising 30 items and a shortened version with 15 items. In the present study, the short version was used, which assesses toxic leadership with five subscales and three items per subscale: Self-promotion, abusive supervision, unpredictability, narcissism, and authoritarian leadership. All items are rated on a 5-point response scale ranging from 1 ‘strongly disagree’ to 5 ’strongly agree’, with subscale scores ranging from 3 to 15. Higher scores indicate greater perceived toxic leadership. The TLS has demonstrated adequate internal consistency in both military and civilian samples (all subscales α > .70), and previous research has supported its five-factor structure [27,28]. In this study, the scale was first translated into Swedish using a back-translation procedure. Minor semantic adjustments to the scale, hereafter referred to as The Toxic Leadership Scale for Sports (TLS-S), were then made to better reflect the language and context relevant to athletes. For example, the phrase ‘My current supervisor’ was replaced with ‘My current head coach.’ An example item is: ’My current head coach publicly belittles team members’ (original: ’Publicly belittles subordinates’). The full set of translated items is provided in S1 Appendix.

### 2.3. Data collection

Data were collected from 01/04/2025–17/12/2025 using a web-based survey administered via the Survey & Report platform. Participants were recruited by convenience sampling through direct contact with representatives of team sports clubs, such as sport directors, head coaches or related staff. The recruitment strategy aimed to include both female and male athletes. Team representatives received both oral and written information detailing the study’s purpose and procedures. The web-based survey was distributed by their coaches to players through a link and QR-code during scheduled team gatherings (e.g., training sessions, travels) or by e-mail, depending on logistical preferences by the team’s coach or sporting director. The survey was administered anonymously to enhance the likelihood of obtaining honest responses. Participants were asked to enter a pre-assigned team code, which enabled an analytical clustering of athletes who belonged to the same team and, by extension, shared the same coach. The team code served no purpose beyond facilitating this analytical clustering.

### 2.4. Statistical analyses

Descriptive statistics (mean and standard deviation (SD)) were used to explore sample characteristics of TLS-S. Intercorrelations between subscales were examined using Spearman rank-order correlations, a non-parametric test appropriate for the ordinal data used in this study. Acknowledging that cutoffs for measures of correlation coefficients strengths need to be interpreted with caution because different guidelines exist [29,30], we followed rule of thumbs suggesting interpretations of coefficients of .10, .20. and .30 as relatively small, typical and relatively large respectively [30]. Scale reliability was assessed using McDonald’s omega (ω_t_) adopting a cutoff of .70 to interpret acceptable internal consistency [31,32]. Omega (ω_t_) has many desirable properties over coefficient alpha as a measure of reliability, such as less stringent and unrealistic assumption concerning, for instance, essential tau-equivalence [33]. Descriptive analyses, non-parametric tests and assessment of scale reliability were performed using SPSS Statistical Package version 30.

**Fig 1:**
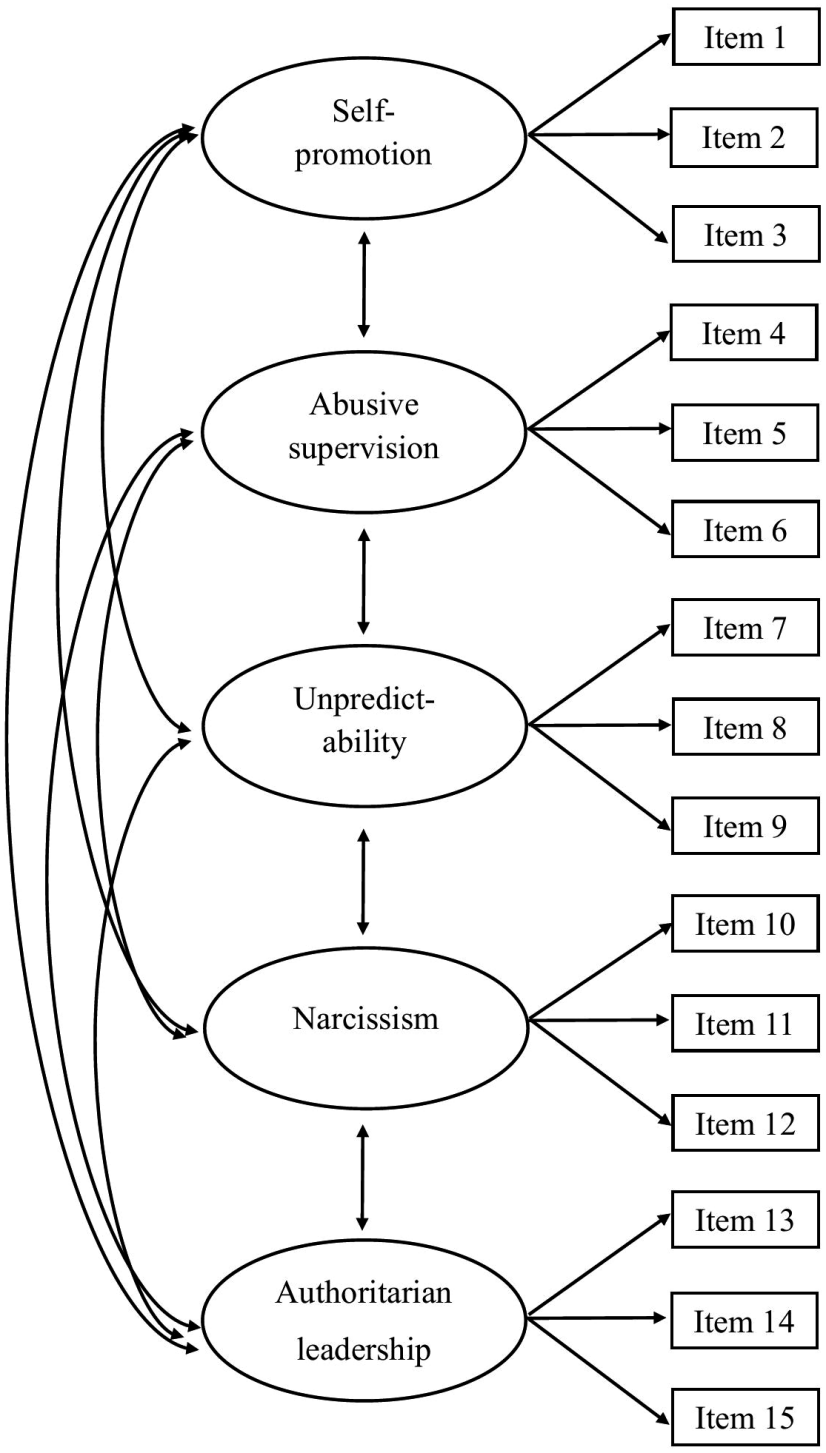
Conceptual model of a first order measurement model with five latent correlated factors.

**Fig 2:**
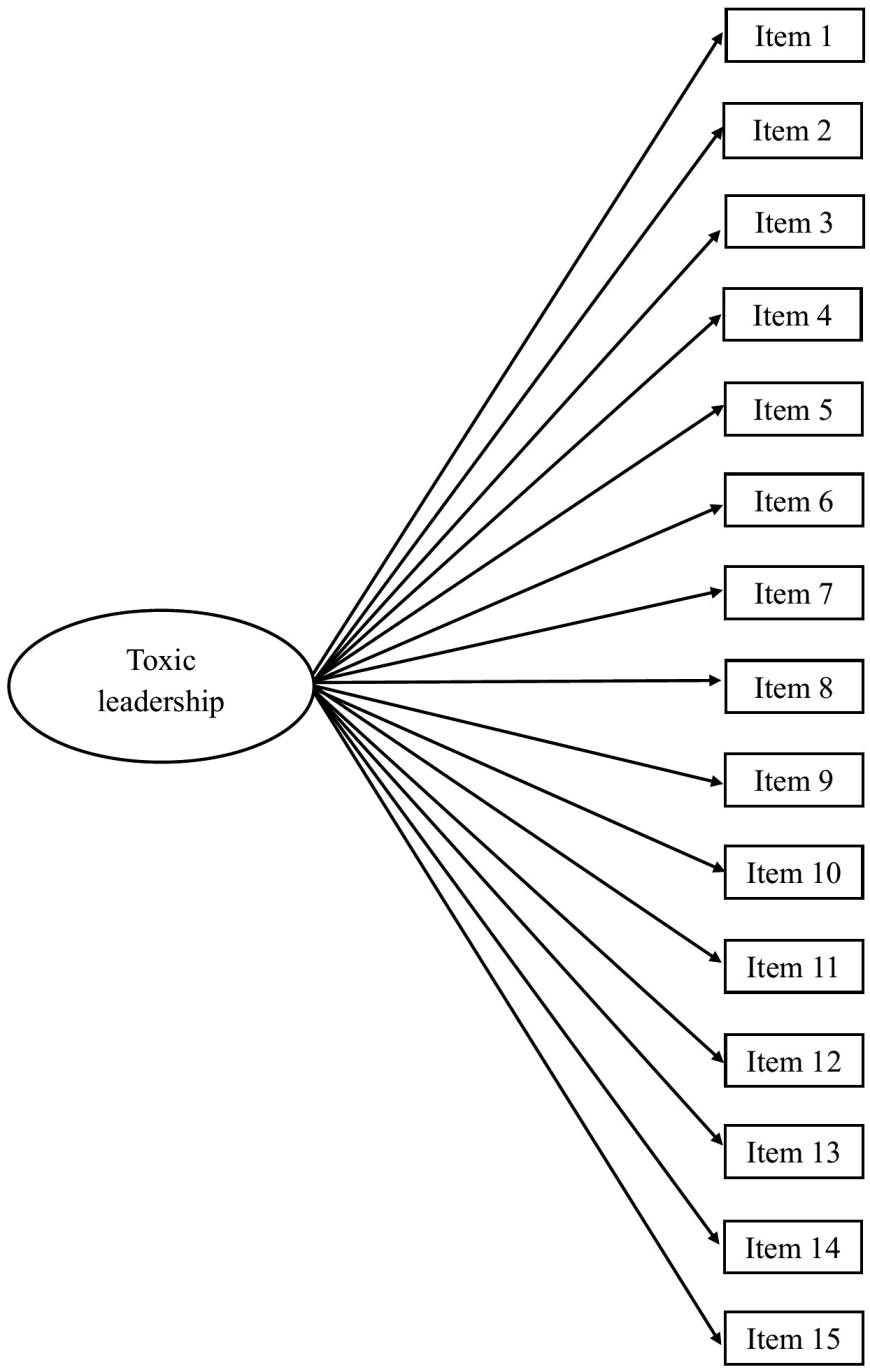
Conceptual model of a first order measurement model with one latent factor.

**Fig 3:**
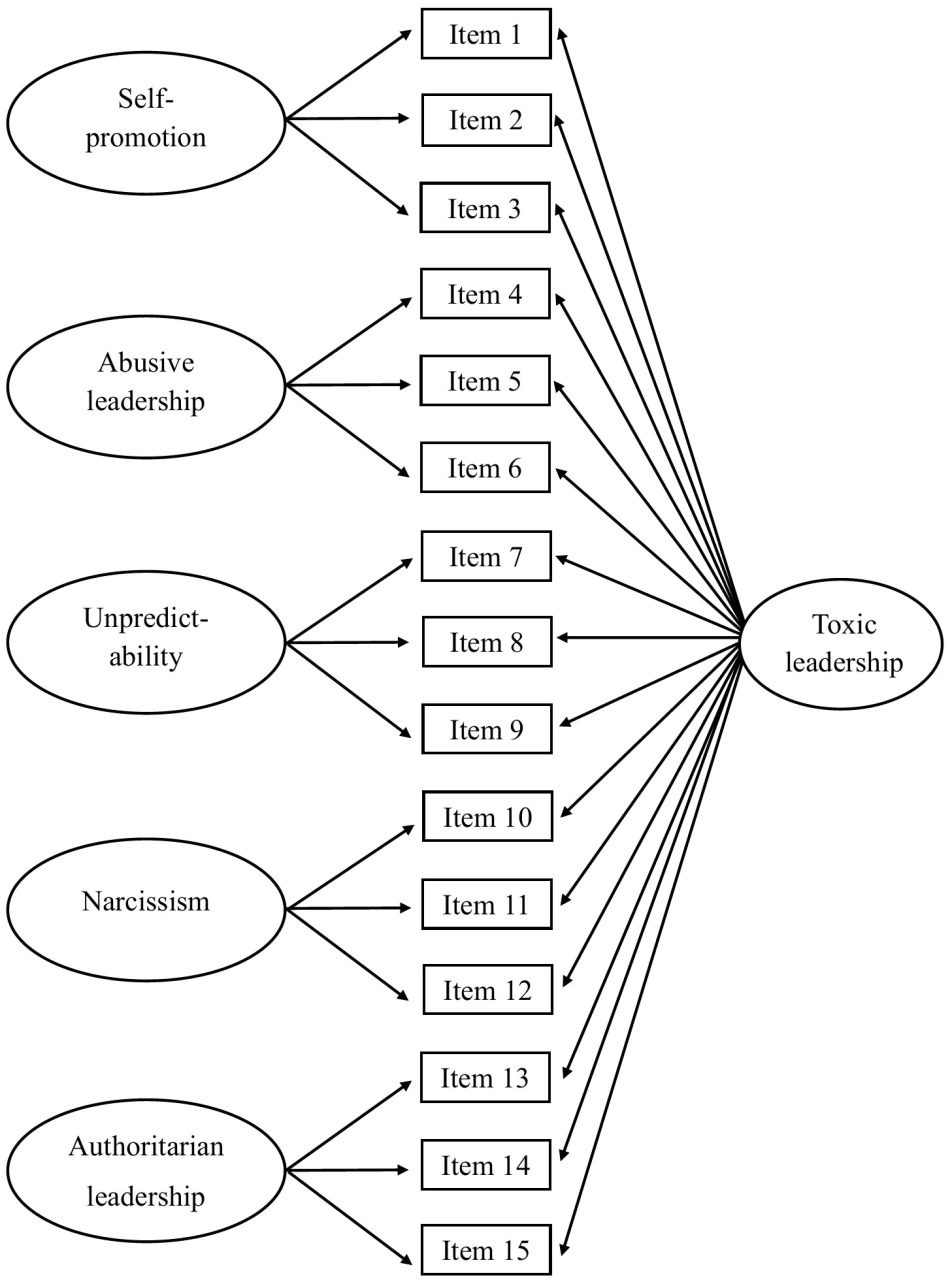
Conceptual model of a bifactor model with one general factor and five latent factors.

Confirmatory factor analyses (CFA) were conducted using MPlus version 9 to investigate the factor structure of three á priori specified models: (a) A first order measurement model with five latent correlated factors (self-promotion, abusive supervision, unpredictability, narcissism, authoritarian leadership; Fig 1), (b) a first order measurement model with one latent factor (Fig 2), (c) a bifactor model with one general factor and five latent factors (Fig 3). The models were hypothesized based on the scale developer’ s original psychometric studies of the TLS [27,28], as well as arguments in the literature that toxic leadership is a cumulative effect of demotivating actions and thereby a unidimensional construct [8,9,14,16,17,21].

Prior to running CFAs, variance inflation factor (VIF) values were examined to diagnose multicollinearity. There are several rule of thumbs discussed to interpret VIF and we adopted the commonly used guideline that VIF values should be < 10 to indicate no serious concerns with multicollinearity [34,35]. The dataset was also screened for multivariate outliers using Mahalanobis distance. Cases were classified as multivariate outliers if they exceeded the chi-square (χ²) threshold corresponding to the appropriate degrees of freedom at a significance level of p < .001 [36]. To mitigate the risk of such outliers exerting undue influence on parameter estimates and compromising overall model fit, identified outliers were removed before conducting the CFAs. To assess the model fit of the hypothesized model, and given the ordinal nature of the data, weighted least squares mean and variance (WLSMV) [37,38] estimation with theta parameterization was used. The WLSMV estimator does not require the assumption of multivariate normality. The CLUSTER command in MPlus was used to model for non-independence in the data related to clusters of respondents having the same coach. Model fit was evaluated using the Comparative Fit Index (CFI) [39] and the Root Mean Square Error of Approximation (RMSEA) [40]. A good model fit is indicated by CFI > .95 and RMSEA < .06. RMSEA values between .08 and .10 indicate a mediocre fit, while values > .10 suggest poor model fit [41]. It should be noted, however, that these conventional cut-off criteria were based on ML-estimation and were not intended to be broadly generalized to models under distinctly different conditions, or estimators, than those in the original study. Furthermore, the idea of one-size-fits-all cut-off criteria for SEM is also a contested topic in the literature [42,43]. Nested models were compared with difference testing described by Muthén and Muthén as appropriate when WLSMV is used as estimator [37]. Statistical significance in all analyses was determined by a p-value < .05.

Explained common variance (ECV) was calculated to determine the extent to which the general factor in the bifactor model dominates relative to the group factors [44,45]. ECV was computed as the proportion of the sum of squared standardized loadings on the general factor relative to the total sum of squared standardized loadings across both the general and group factors [45]. A higher ECV value (>.70) indicates that the general factor accounts for most of the common variance, supporting essential unidimensionality. Conversely, lower ECV values suggest that specific factors explain a greater proportion of variance, indicating a multidimensional structure [44,45]. Percentage of uncontaminated correlations (PUC) was also calculated to complement ECV by considering the overall data structure. PUC represents the proportion of item correlations that are unaffected by shared group factors. PUC tends to be higher when there are many group factors with relatively few items per factor, compared to situations where there are fewer group factors but a larger number of items within each factor. When both ECV (>.70) and PUC (>.70) are high, the common variance can be interpreted as essentially unidimensional, with only minor bias introduced by group factors [45]. Missing data in all analyses were handled using pairwise deletion.

### 2.5. Ethical statement

The study was submitted to the Swedish Ethical Review Authority for ethical approval. According to Swedish ethical rules, parental consent is required for children and young people aged 14 or younger. Based on the study’s design, where all participants were aged 15 years or older and the survey was conducted anonymously without collecting any personal data that could reveal the identity of the participants, the Swedish Ethical Review Authority evaluated that the study did not fall within the scope of the Swedish Ethical Review Act and therefore was not in need of formal ethical approval by the Swedish Ethical Review Authority (Dnr: 2025-00838-01). At the beginning of the survey, all participants were presented with written information about the study to enable an informed decision regarding their participation. Written informed consent was obtained through the first question of the survey, in which participants were asked whether they consented to take part in the study. Those who declined to provide consent at this stage were unable to proceed, as the survey was automatically terminated.

## 3. Results

### 3.1. Descriptives of the TLS-S, internal consistency and subscale intercorrelations

Descriptive statistics (mean, SD) for the subscales of the TLS-S, along with intercorrelations, internal consistency estimates as well as skewness and kurtosis, are presented in Table 1. Spearman rank-order correlations revealed relatively large associations among the subscales (self-promotion, abusive supervision, unpredictability, narcissism, authoritarian leadership) in the TLS-S. The authoritarian leadership subscale exhibited the weakest intercorrelations with the other subscales. Internal consistency, assessed using McDonald’s omega, indicated satisfactory reliability for four subscales as well as the TLS-S total score (ωₜ > .70). In contrast, the authoritarian leadership subscale (ωₜ = .61) fell below the accepted threshold, indicating inadequate reliability. Item-level response distributions for the TLS-S, grouped by gender and sports, are presented in S2 Appendix. Partitioning the sample into subsamples resulted in small groups and item response patterns revealed that certain scale points were not utilized within some subsamples, likely due to these small sizes. Therefore, at this initial stage of psychometric evaluation of the TLS-S, we decided to conduct all subsequent analyses on the total sample.

**Table 1:** Descriptive statistics, intercorrelations and internal consistency for the original subscales of the TLS-S.

Subscale TLS-S	Spearman rank order correlations				McDonald’s Ѡt	Mean (SD)	Skewness	Kurtosis
	2.	3.	4.	5				
Self-promotion (score: 3-15)	.59* (n=187)	.53* (n=188)	.57* (n=183)	.40* (n=184)	.79	5.19 (2.57)	1.40	1.93
Abusive supervision (score: 3-15)	--	.57* (n=187)	.62* (n=182)	.44* (n=183)	.78	5.18 (2.45)	1.49	2.34
Unpredictability (score: 3-15)	--	--	.70* (n=183)	.42* (n=184)	.72	5.89 (2.57)	1.01	.77
Narcissism (score: 3-15)	--	--	--	.53* (n=181)	.86	5.74 (2.75)	1.02	.67
Authoritarian leadership (score: 3-15)	--	--	--	--	.61	6.72 (2.46)	.66	.21
TLS-S Total score (score: 15-75)	--	--	--	--	.90	28.58 (10.56)	1.08	1.20

Note. * p < .001

**Table 1. Descriptive statistics, intercorrelations and internal consistency for the original subscales of the TLS-S.**

### 3.2. Confirmatory factor analyses

**Fig 4:**
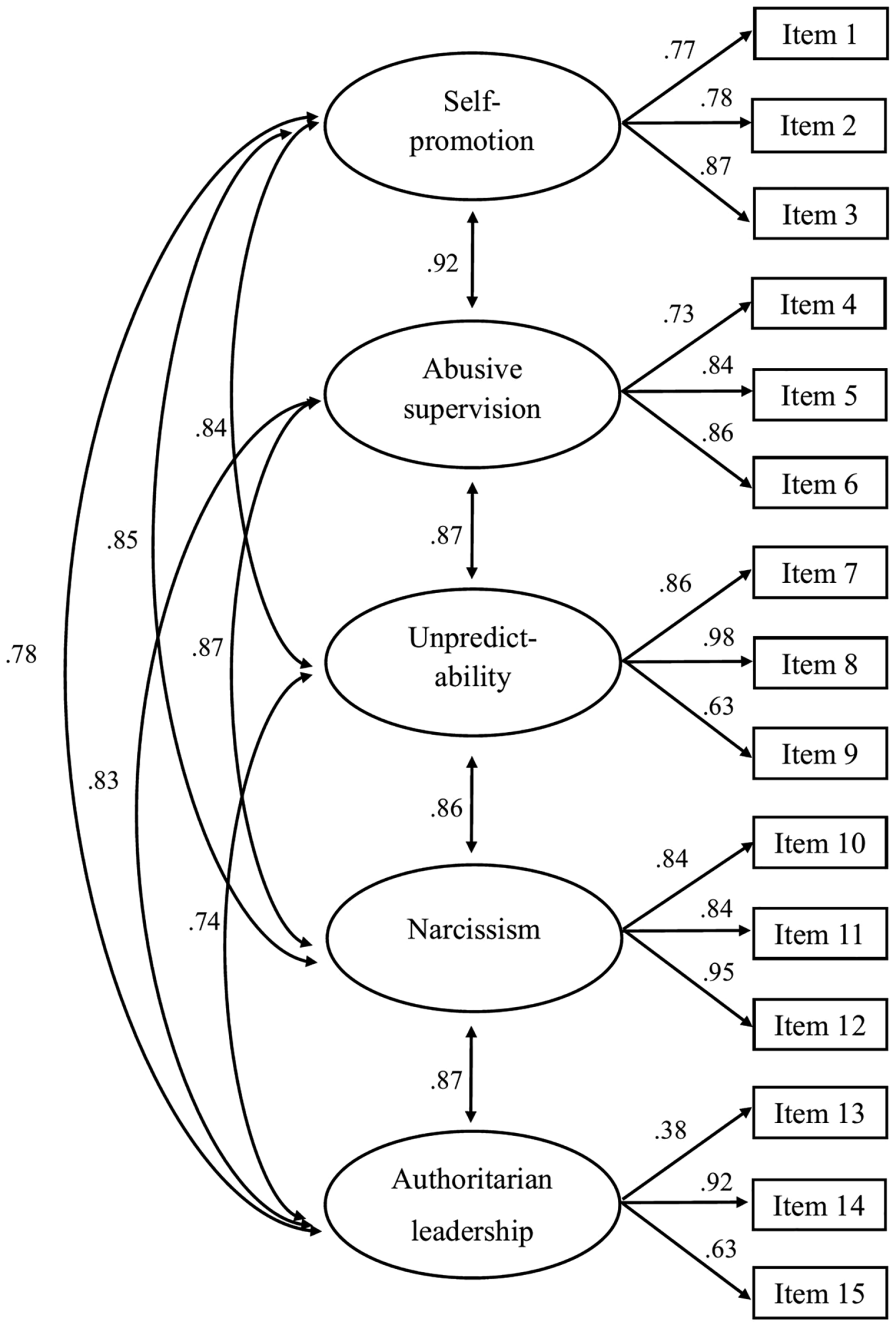
Confirmatory factor analysis with the five-factor correlated measurement model (standardized factor loadings).

Multicollinearity was not identified as a problem in the data, as VIF values were below 10 for all items (range: 1.23–3.01). Mahalanobis distance identified eight multivariate outliers (χ^2^(15) = 37.7, p  < .001), which were removed before CFAs were performed. Thus, a final sample of 181 cases was used for CFA analyses. Although both the five-factor and the one-factor model overall exhibited acceptable fit indices, indices favored the five-factor solution (Table 2). The difference test for WLSMV confirmed that the five-factor model provided significantly better model fit than the one-factor solution (p < .001). As shown in Fig 4, all standardized factor loadings in the five-factor model exceeded .60, except for item 13, which had a lower loading (.38). This finding suggests that item 13 may not represent the underlying construct (i.e., authoritarian leadership) as strongly as other items on the subscale. Intercorrelations among the five factors were high (range: .73−.92), indicating strong associations between the five subscales (Fig 4). The bifactor model demonstrated good overall fit to the data. Because this specification was not nested within the alternative models, statistical difference testing was not applicable. Instead, model adequacy was evaluated descriptively through fit indices, which supported the appropriateness of the bifactor structure.

**Table 2:** Confirmatory Factor Analysis and χ² Difference Testing for Nested Model Comparison.

Model		Χ^2^	df	CFI	RMSEA (90% CI)	SRMR	Χ^2^ test for Difference testing	Df difference	p
1.	Five-factor model	135.87*	80	.98	.062 (.044−.080)	.044	--		--
2.	One-factor model	172.46*	90	.97	.071 (.055−.087)	.052	53.60	10	<.001
3.	Bifactor model	142.72*	81	.98	.065 (.047−.082)	.050	Not nested	--	--

Note. * p < .001

Standardized factor loadings and item-level explained variance (R^2^) for the bifactor model are presented in Table 3. Overall, items exhibited stronger loadings on the general factor compared to the subscales. Two exceptions emerged: Item 13 loaded more strongly on the authoritarian leadership subscale than on the general factor, and item 1 showed comparable factor loadings on the general factor and the self-promotion subscale. The computed explained common variance (ECV) was .79 and the percentage of uncontaminated correlations (PUC) was .86. Together, the high values (>.70) of both ECV and PUC suggest that most variance in the TLS-S are primarily explained by the general factor and that most item correlations are unaffected by shared group factors, supporting the model’s unidimensional nature.

**Table 3:** Standardized Factor Loadings and Item-Level Explained Variance (R²) in the Bifactor Model.

	General factor: Toxic leadership		Self- promotion		Abusive supervision		Unpredict-ability		Narcissism		Authoritarian leadership	
Item	Factor loading	R^2^	Factor loading	R^2^	Factor loading	R^2^	Factor loading	R^2^	Factor loading	R^2^	Factor loading	R^2^
1.	.58	.33	.58	.33	--	--	--	--	--	--	--	--
2.	.71	.51	.38	.14	--	--	--	--	--	--	--	--
3.	.79	.63	.33	.11	--	--	--	--	--	--	--	--
4.	.73	.54	--	--	.48	.23	--	--	--	--	--	--
5.	.81	.66	--	--	−.06	.00	--	--	--	--	--	--
6.	.83	.69	--	--	−.22	.05	--	--	--	--	--	--
7.	.77	.60	--	--	--	--	.45	.20	--	--	--	--
8.	.89	.79	--	--	--	--	.40	.16	--	--	--	--
9.	.59	.35	--	--	--	--	−.17	.03	--	--	--	--
10.	.78	.61	--	--	--	--	--	--	.44	.20	--	--
11.	.79	.62	--	--	--	--	--	--	.38	.14	--	--
12.	.90	.81	--	--	--	--	--	--	.17	.03	--	--
13.	.32	.11	--	--	--	--	--	--	--	--	.67	.45
14.	.81	.66	--	--	--	--	--	--	--	--	.24	.06
15.	.56	.32	--	--	--	--	--	--	--	--	.28	.08
Sum R^2^:	--	8.22	--	.59	--	.28	--	.39	--	.37	--	.58

## 4. Discussion

The present study assessed the psychometric properties of the Toxic Leadership Scale adapted for sport settings (TLS-S). Overall, the findings supported the reliability and the factorial validity of the TLS-S within a team sport context. Internal consistency was satisfactory (ωₜ > .70) for the total TLS-S score and four of the five subscales: self-promotion, abusive supervision, unpredictability, and narcissism. The authoritarian leadership subscale demonstrated weaker reliability, with item 13 (‘My current head coach controls how team members complete their tasks’) also exhibiting a high degree of uniqueness. Users of the scale should be attentive to this issue, and interpret particularly item 13 with caution. If similar patterns are observed in future research, this item should be considered for removal or reformulation to more accurately reflect authoritarian leadership in sport settings. The findings provided initial evidence for the factorial validity of the TLS-S. While all three tested models (i.e., the five-factor model, the one-factor model, the bifactor model) demonstrated acceptable model fit, the subscales displayed high intercorrelations. Results from the bifactor model provided evidence for a predominantly unidimensional structure of the TLS-S. The general factor accounted for the majority of variance, suggesting that toxic leadership, as assessed by the TLS-S, is appropriately conceptualized as a single overarching construct.

Researchers have debated whether destructive leadership (e.g., abusive supervision, narcissism, authoritarianism) should be treated as distinct constructs or as overlapping dimensions within a unified framework, with arguments supporting both views [18,46]. Differentiating between specific forms of destructive leadership may enable more nuanced analyses and targeted interventions; however, empirical evidence suggests that leaders’ traits or behaviors may not, in isolation, be perceived by followers as inherently toxic. For example, leaders exhibiting authoritarian tendencies, characterized by unilateral decision-making, strict discipline, and expectations of unquestioning obedience [47], or narcissistic traits such as self-centeredness and grandiosity [48], have traditionally been regarded as less effective or “dark” due to their controlling and autonomy-suppressive characteristics [49]. Yet, contemporary research indicates that neither authoritarian nor subclinical narcissistic tendencies alone are necessarily destructive or harmful to followers. Authoritarian leadership has been associated with both positive and negative outcomes depending on group context and objectives [47]. Similarly, studies on subclinical narcissism and leadership effectiveness report mixed findings. A systematic review [49] revealed a curvilinear relationship between narcissism and leadership effectiveness, suggesting that moderate levels of narcissism may be beneficial. Nevertheless, narcissistic leaders often engage in self-enhancement, particularly when outcomes are assessed through self-report measures, raising concerns about the reliability and validity of such data [49]. In performance-driven environments such as elite sports, perceived coach narcissism may even be associated with outcomes athletes regard as advantageous [50,51]. Specifically, athletes may interpret narcissistic traits in coaches as beneficial, as these traits can reinforce athletes’ self-enhancement motives and create opportunities to showcase their skills during competition [50].

Our findings support previous scholars who have suggested that toxic leadership should be investigated as a holistic construct [7,14,16–22]. Arguably, athletes are likely to respond to leadership measures from judgments about their coach across multiple situations over time. Rather than differentiating between clearly delineated and distinct leadership behaviors or traits, athletes may integrate diverse cues into a general perception of whether the coach is toxic. Moreover, assessing toxic leaders solely through their characteristics and traits without considering cultural and system-related factors remains incomplete, as it neglects the influence of follower behaviors and organizational culture in enabling or amplifying toxicity [14,19,21–23]. Given the self-serving motives and behavioral patterns inherent in toxic leadership, such leaders can, however, be difficult to detect because they often conceal harmful actions from individuals perceived as influential or capable of advancing their careers [7,14,20,21]. Lipman-Blumen [20] contended that, although single whistleblowers may occasionally try to disclose such leaders, most followers, even in the presence of highly toxic leadership, typically remain silent and passive due to psychological, existential, financial, political, and social barriers to disengagement. As a result, followers may endure extended periods of inaction, anticipating that others will ultimately hold toxic leaders accountable. Meanwhile, toxic leaders can continue to perpetuate reassuring illusions of omnipotence and benevolence, presenting themselves as indispensable saviors, even as the consequences of their actions generate diverse forms and degrees of harm for both individuals and organizations [20].

The prevailing performance-oriented culture in high-performance sports, where funding and future opportunities for athletes, coaches, and organizations are closely tied to demonstrated status and medal counts [3,52,53], may also serve to excuse, normalize, or even incentivize toxic leadership within sports organizations. Although toxic leaders may deliver short-term results, any gains typically occur at the long-term expense of athletes’ wellbeing, impaired interpersonal relationships, and organizational stability [14]. Consequently, prioritizing short-term funding or medal objectives above all else may inadvertently create conditions that legitimize harmful leadership practices and undermine both long-term athlete development and organizational integrity. For organizations, such prioritization could therefore constitute a form of self-destructive behavior, whereby perceived short-term benefits ultimately lead to long-term adverse consequences. Furthermore, unquestioned and empirically unsubstantiated assumptions regarding the necessity of harsh, tough and controlling leadership to foster athletic success can produce further systemic vulnerabilities and a notably resistance to change within sports organizations [54]. When core beliefs are reinforced by unspoken traditions, individuals and organizations often cling to outdated practices, assuming the status quo is both optimal and necessary. This mindset can foster resistance to new ideas and methods, ultimately hindering development and adaptation to norms supported by research as enhancing both health and performance over the long term [54]. Once toxic dynamics become entrenched, they are exceedingly difficult to disrupt, as the underlying processes may become self-reinforcing and perpetuate themselves by attracting and socializing new leaders who adopt similar values and toxic behavioral patterns. Early identification, for example through regular and systematic assessments of both the constructive and destructive dimensions of leadership, and proactive measures to curb the development of toxic leadership from the outset are therefore essential [3].

While this study represents a novel effort to facilitate the assessment of toxic leadership within sport contexts, several limitations nevertheless warrant consideration. First, the sample size was relatively small and consisted exclusively of athletes from team sports, which restricts the generalizability of the findings. Moreover, teams included in this study had predominantly male coaches. Although this distribution reflects the gender composition typically observed among elite-level coaches, it is important to note that the athletes’ evaluations primarily pertain to male coaches which may introduce bias in the results. The sample size in this study did not allow for subgroup analyses, such as tests of measurement invariance across gender or sport type. We encourage researchers to pursue this work in future studies. Given the ordinal nature of the data, we employed WLSMV as the estimation method, as it typically yields less biased parameter estimates compared to alternatives such as robust maximum likelihood (MLR). However, prior research indicates that WLSMV can moderately overestimate interfactor correlations when sample sizes are small [55]. Although model fit in this initial validation study was adequate, replication in larger samples is recommended. Second, the study population was drawn from a single Scandinavian country. Culture may influence athletes’ perceptions of leadership, including which behaviors are regarded as effective or detrimental. These cultural nuances highlight the need for future research to evaluate the scale across diverse cultural settings. Such efforts would strengthen understanding of its relevance, applicability, and potential for cross-cultural comparisons among athletes, coaches, managers, stakeholders, and other members of the sporting entourage in varied national contexts. Third, in performance-oriented cultures, toxic leadership and even abusive behaviors can become normalized. Despite their inherently destructive nature, such behaviors may be perceived as acceptable or even encouraged within these environments [3–5]. Consequently, individuals completing self-report scales may struggle to identify themselves as followers of toxic leaders. Awareness of these harmful dynamics often emerges only retrospectively, once individuals have exited the environment and gained critical distance [3], This represents a general limitation of self-report instruments and poses a risk of systematic underreporting of toxic leadership, even in contexts where it is highly prevalent. It is also plausible that individuals exhibiting toxic leadership tendencies may decline participation in leadership studies, thereby introducing sampling bias in research reliant on voluntary participation. In the present study, we used convenience sampling, and thus we cannot exclude the possibility that our findings were influenced by sampling bias. To estimate the prevalence of toxic leadership in future investigations, the use of random probability sampling is strongly recommended.

### 5. Conclusions

In conclusion, the TLS-S is a promising instrument for assessing toxic leadership within team sport contexts. Our results provide support for the scale’s internal consistency and for a unidimensional factor structure. This study represents a novel contribution to the quantitative assessment of toxic leadership in sport and establishes a foundation for subsequent research aimed at further validation and systematic refinement of the scale across more diverse athletic populations and cultural contexts.

## Supporting information

S1 Appendix The Toxic Leadership Scale for Sports (TLS-S). The Swedish translation the Toxic Leadership Scale adapted to sports (TLS-S) and a preliminary English version.
(DOCX)

S2 Appendix Item-level response patterns of the TLS-S. Item-by-item analysis of the TLS‑S by gender and sports.
(DOCX)
