# Supplementary material for: Assessing toxic leadership in high-performance sports: Psychometric validation of a Swedish Version of the Toxic Leadership Scale for Sports (TLS-S)
Source: PLoS One. 2026 Mar 6;21(3):e0343533. doi: 10.1371/journal.pone.0343533 (PMC12965676; doi:10.1371/journal.pone.0343533)
Supplement: S1 Appendix — The Swedish translation the Toxic Leadership Scale adapted to sports (TLS-S) and a preliminary English version. (DOCX) [file pone.0343533.s001.docx]

| **English original 15-item version of TLS**  **(Schmidt, 2008)** | | **Swedish translation and sports modification TLS-S (Preliminary English version)** | |
| --- | --- | --- | --- |
| *My current supervisor…* | | *Min nuvarande huvudtränare…*  *(My current headcoach...)* | *Subscale* |
| 1. | Drastically changes his/her demeanor when his/her supervisor is present | Ändrar sitt beteende drastiskt när hans/hennes överordnade är närvarande (Drastically changes his/her behavior when his/her supervisor is present) | Self-promotion |
| 2. | Will only offer assistance to people who can help him/her get ahead | Erbjuder endast hjälp till personer som kan hjälpa honom/henne att lyckas (Offer only assistance to people who can contribute to his/her success) | Self-promotion |
| 3. | Accepts credit for successes that do not belong to him/her | Tar åt sig äran för framgångar som inte tillhör honom/henne  (Takes credit for successes that do not belong to him/her) | Self-promotion |
| 4. | Holds subordinates responsible for things outside their job descriptions | Håller lagmedlemmar ansvariga för saker som ligger utanför deras uppgifter  (Holds team members responsible for things outside their assignments) | Abusive supervision |
| 5. | Publicly belittles subordinates | Förlöjligar lagmedlemmar offentligt (Publicly belittles team members) | Abusive supervision |
| 6. | Reminds subordinates of their past mistakes and failures | Påminner lagmedlemmar om deras tidigare misstag och misslyckanden  (Reminds team members of their past mistakes and failures) | Abusive supervision |
| 7. | Allows his/her current mood to define the climate of the workplace | Låter sitt humör avgöra stämningen i laget (Allows his/her current mood to define the climate of the team) | Unpredictability |
| 8. | Expresses anger at subordinates for unknown reasons | Uttrycker ilska mot lagmedlemmar av oklara skäl  (Expresses anger at team members for unknown reasons) | Unpredictability |
| 9. | Varies in his/her degree of approachability | Varierar i sin grad av tillgänglighet  (Varies in his/her degree of approachability) | Unpredictability |
| 10. | Has a sense of personal entitlement | Visar en attityd av berättigande över andra (Displays an attitude of entitlement over others) | Narcissism |
| 11. | Thinks that he/she is more capable than others | Anser att han/hon är mer kompetent än andra  (Thinks that he/she is more capable than others) | Narcissism |
| 12. | Believes that he/she is an extraordinary person | Uppträder som att han/hon är en extraordinär person  (Acts as if he/she is an extraordinary person) | Narcissism |
| 13. | Controls how subordinates complete their tasks | Kontrollerar hur lagmedlemmar utför sina uppgifter  (Controls how team members complete their tasks) | Authoritarian leadership |
| 14. | Does not permit subordinates to approach goals in new ways | Tillåter inte lagmedlemmar att närma sig mål på nya sätt  (Does not permit team members to approach goals in new ways) | Authoritarian leadership |
| 15. | Determines all decisions in the unit whether they are important or not | Fattar alla beslut i ledargruppen, oavsett om de är viktiga eller inte  (Determines all decisions in the leader group whether they are important or not) | Authoritarian leadership |

Reference:

Schmidt, A. A. Development and validation of the toxic leadership scale. University of Maryland, 2008. Available at: https://scispace.com/pdf/development-and-validation-of-the-toxic-leadership-scale-2dr91qipqn.pdf
