# Supplementary figures and images for: Assessing toxic leadership in high-performance sports: Psychometric validation of a Swedish Version of the Toxic Leadership Scale for Sports (TLS-S)
Source: PLoS One. 2026 Mar 6;21(3):e0343533. doi: 10.1371/journal.pone.0343533 (PMC12965676; doi:10.1371/journal.pone.0343533)

***Gender***


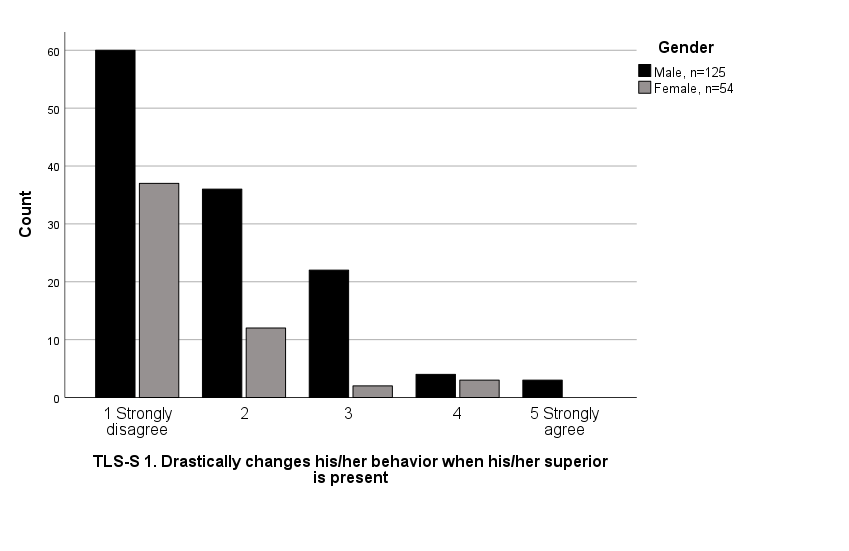


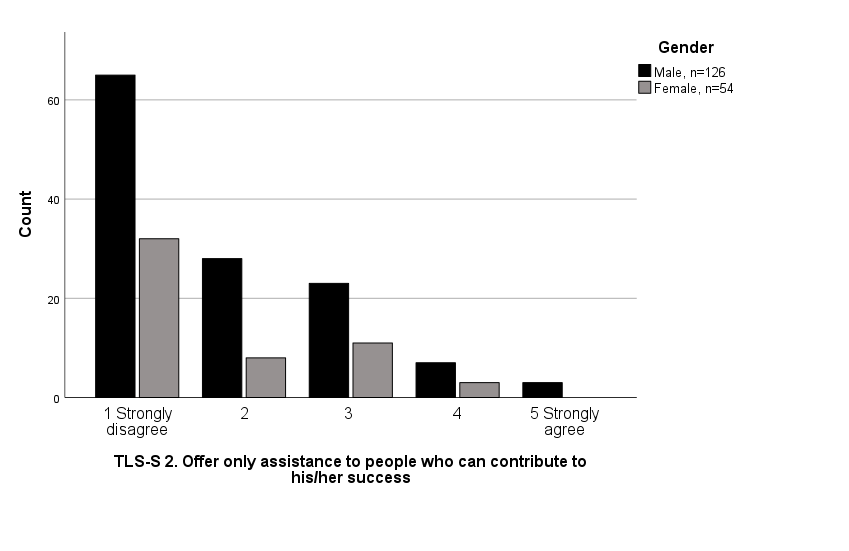


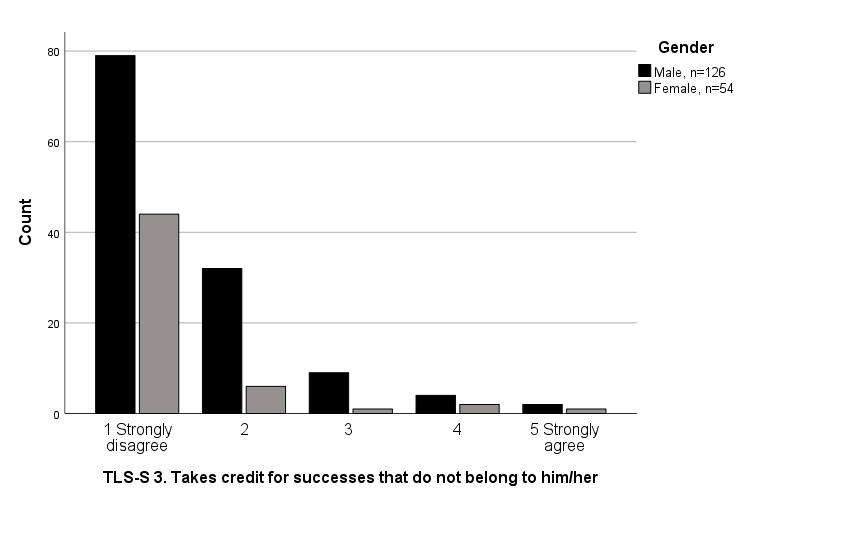


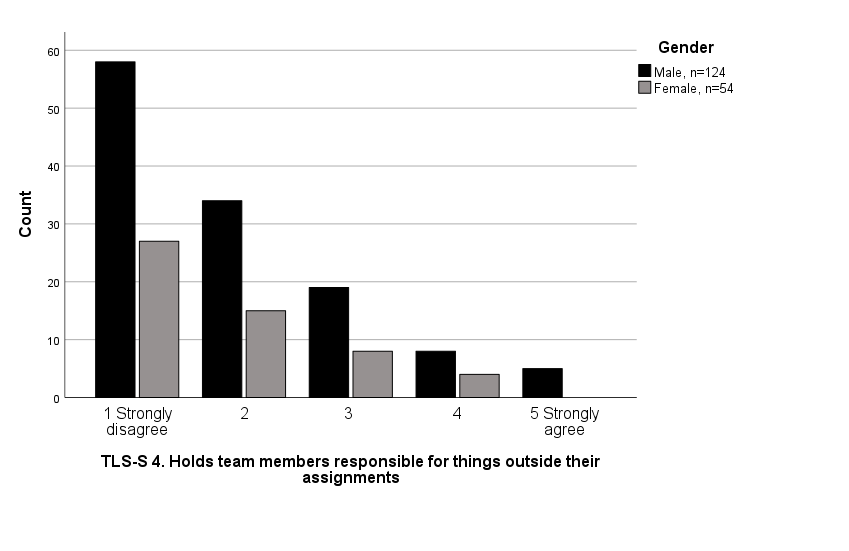


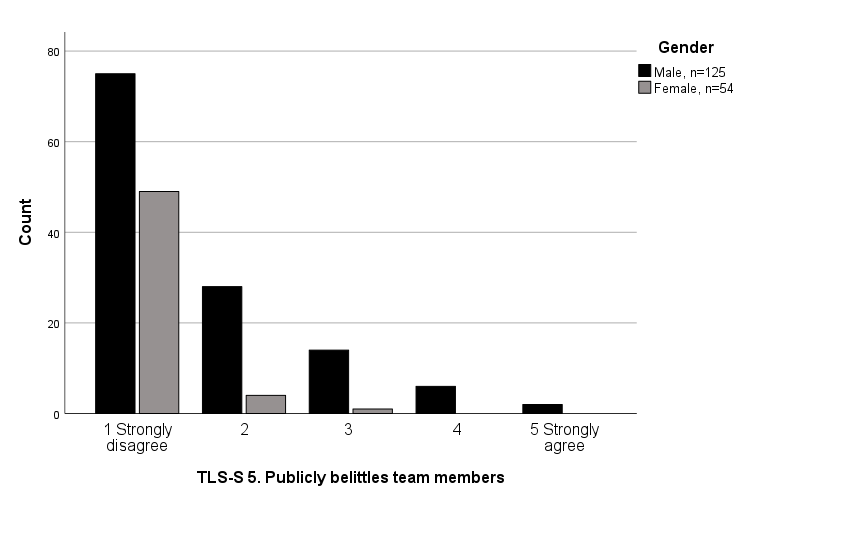


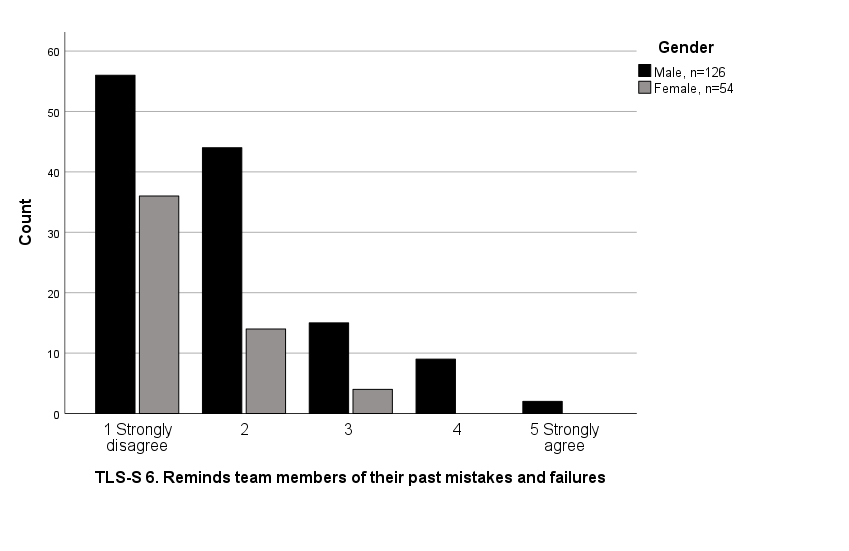


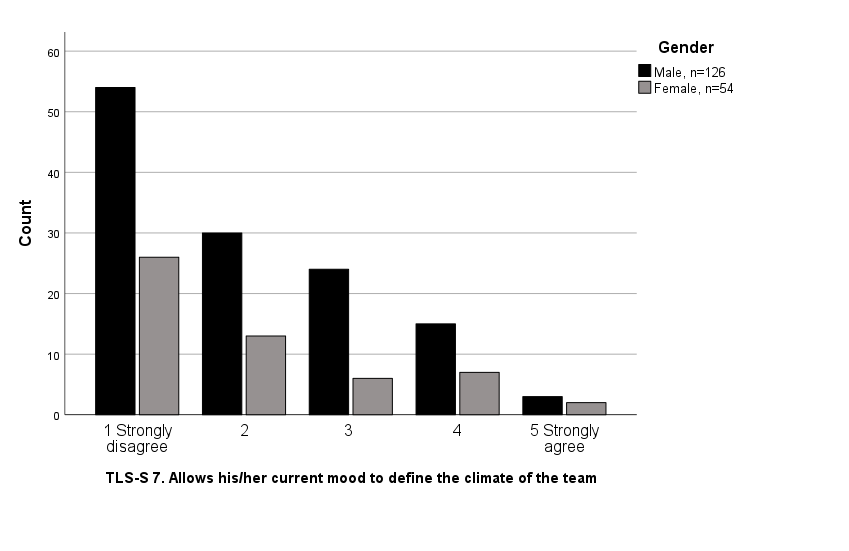


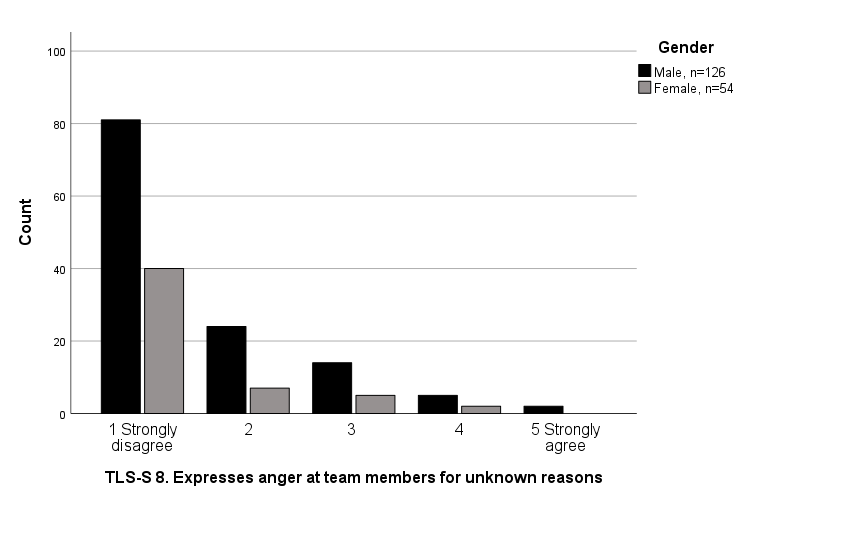


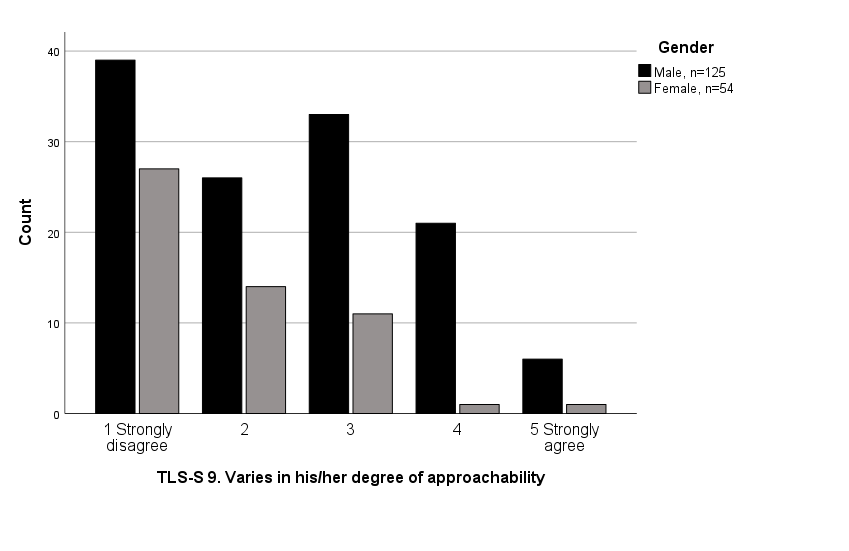


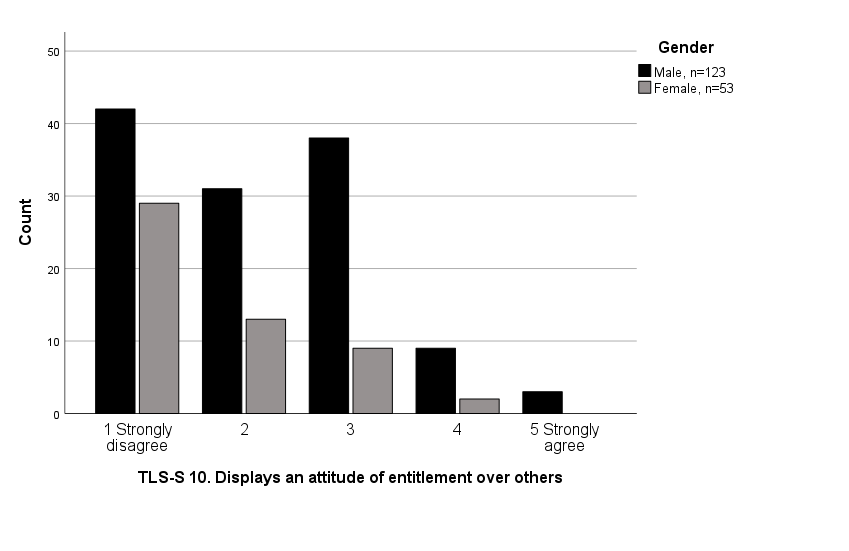


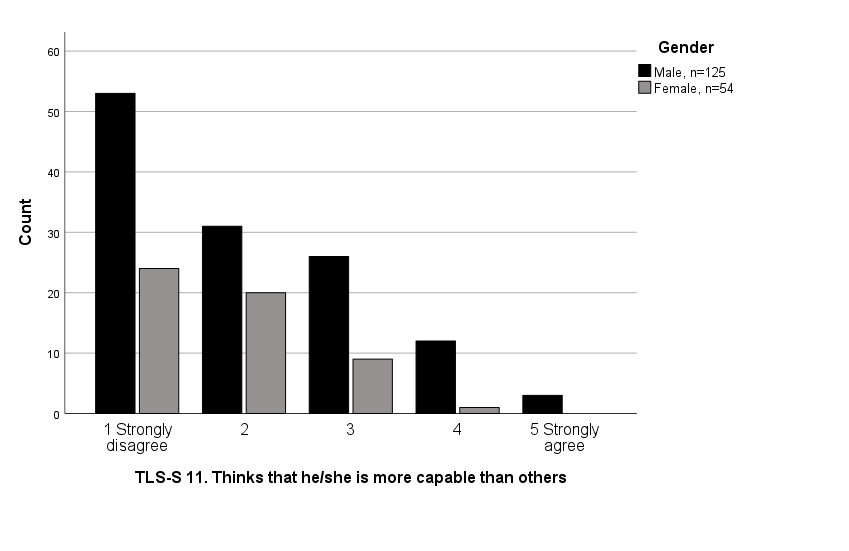


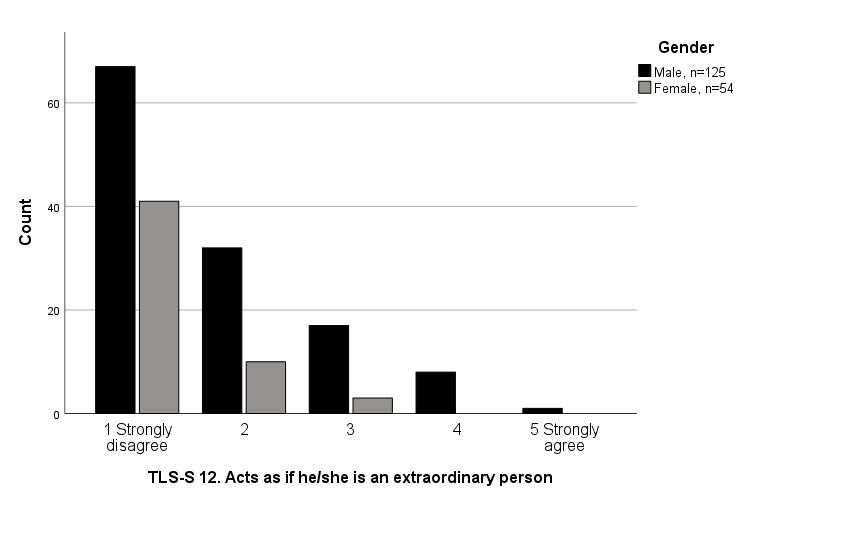


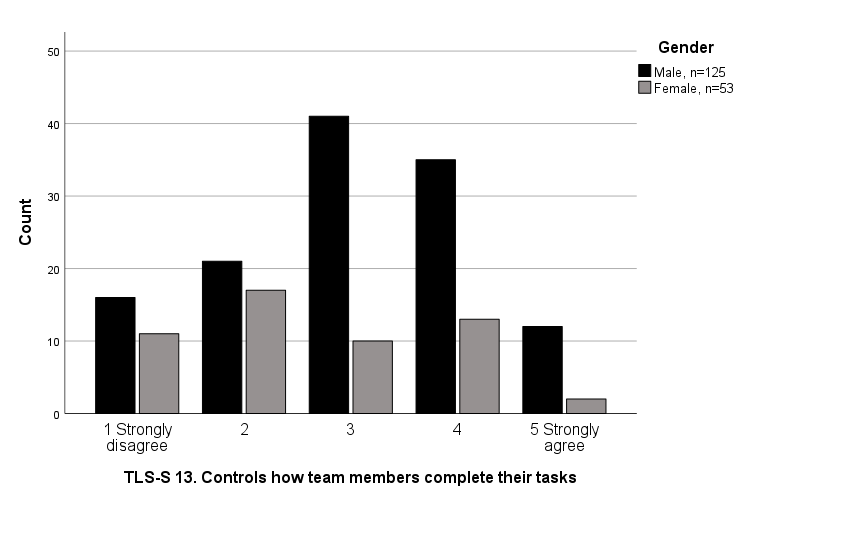


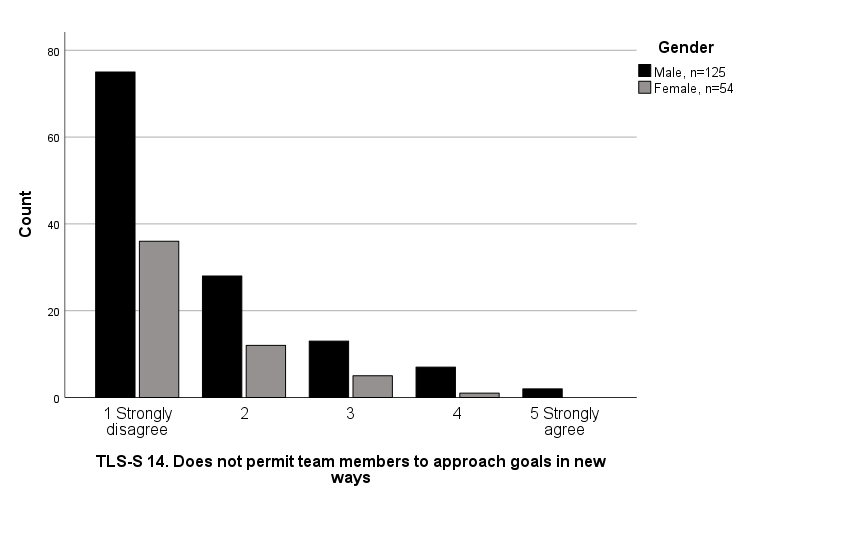


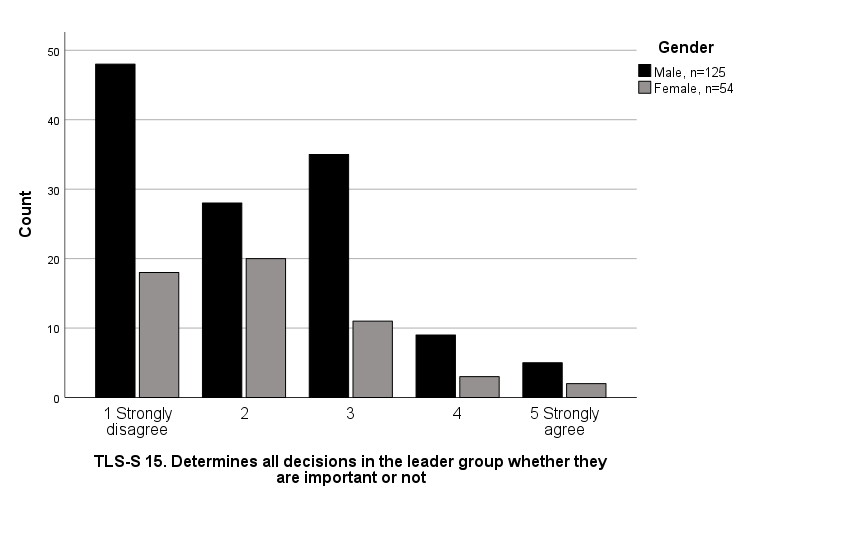


***Sports*** (sports with <5 participants not reported)

**
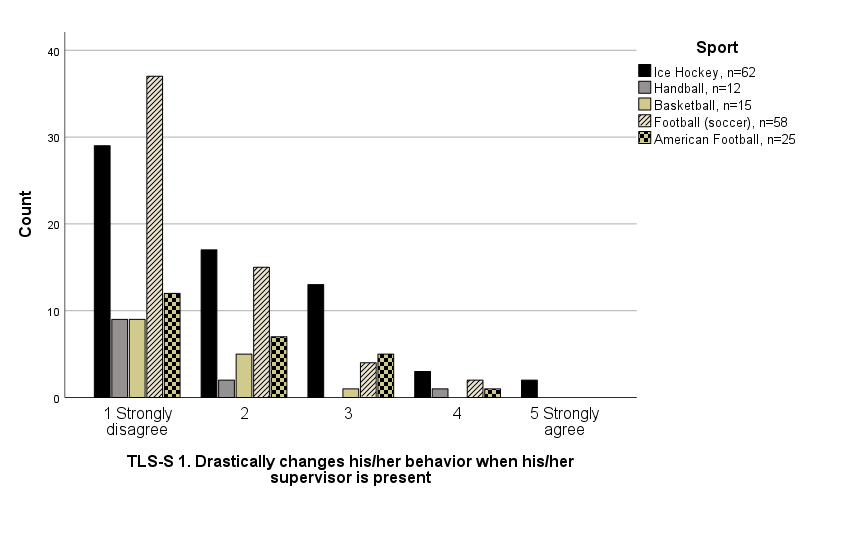
**

**
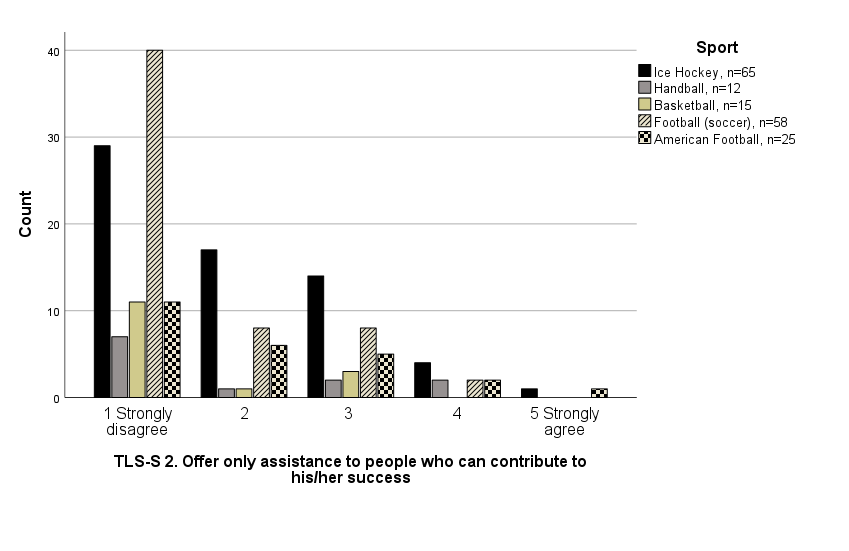
**

**
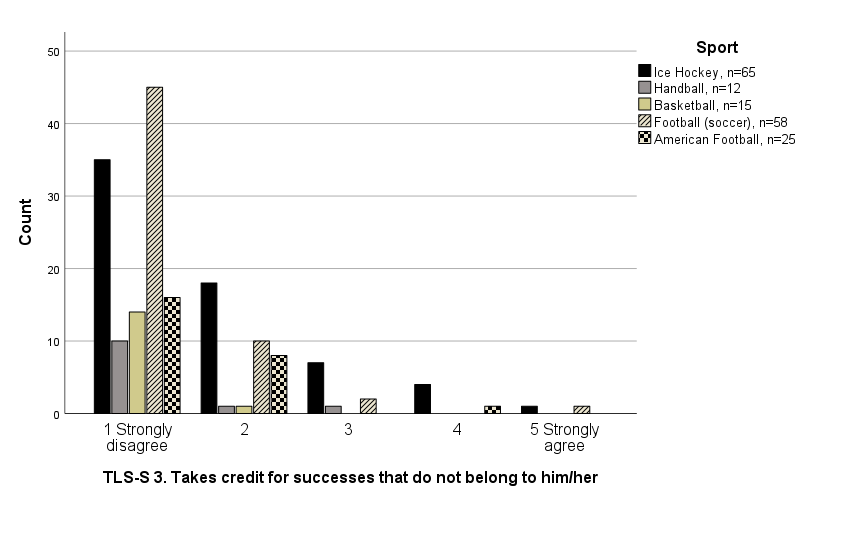
**

**
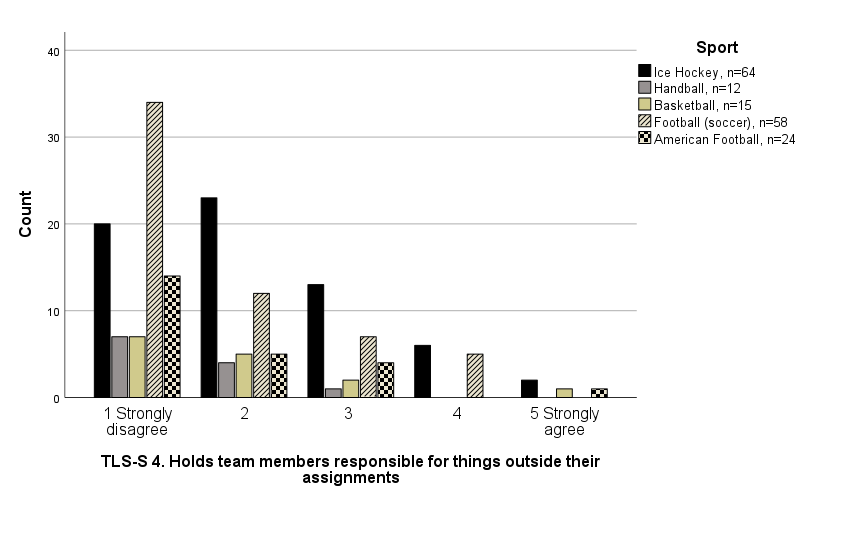
**

**
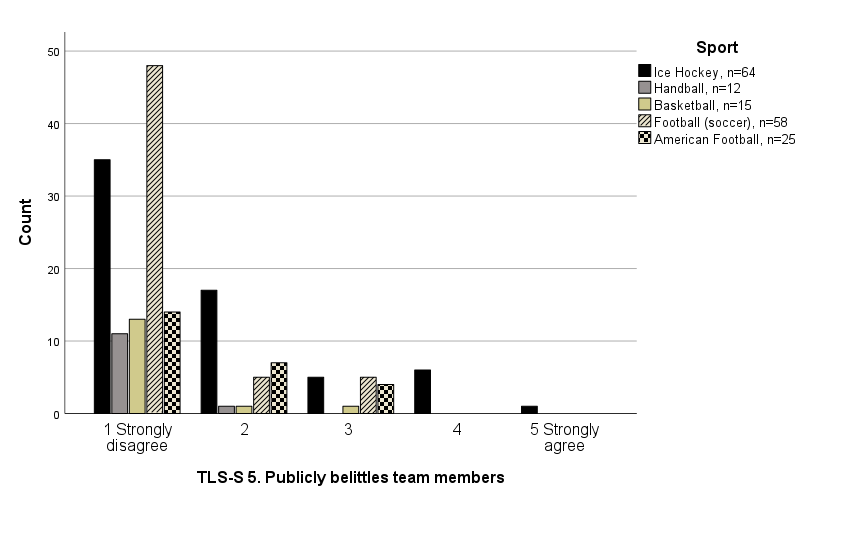
**

**
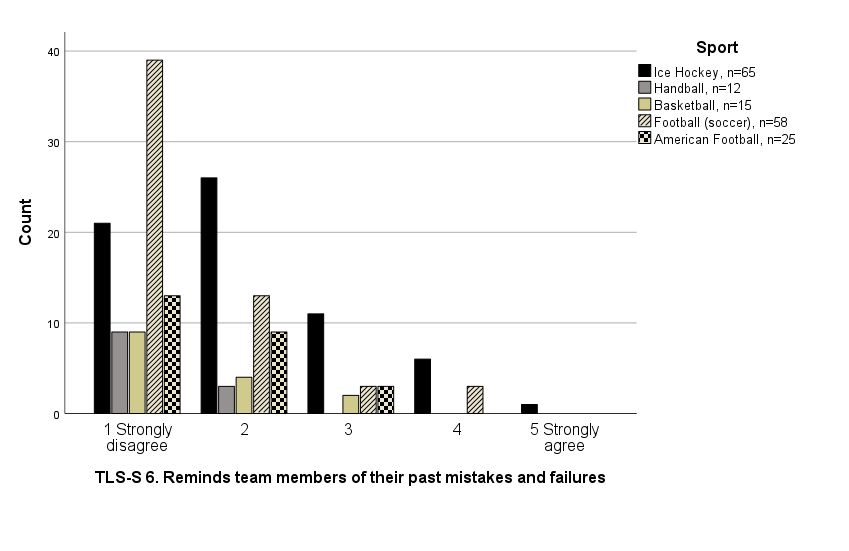
**

**
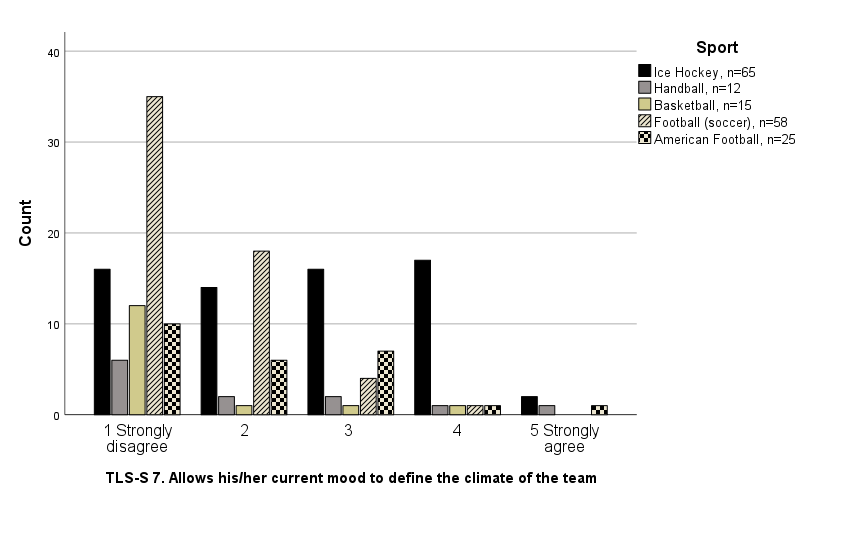
**

**
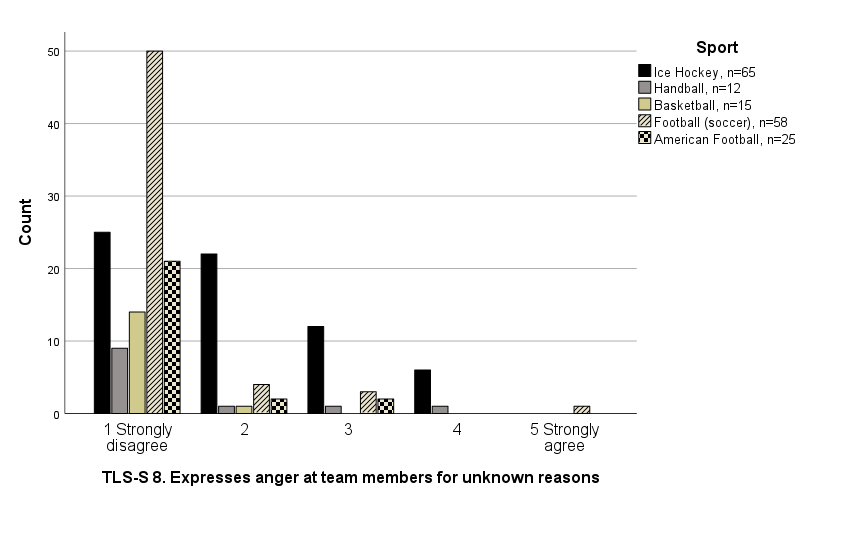
**

**
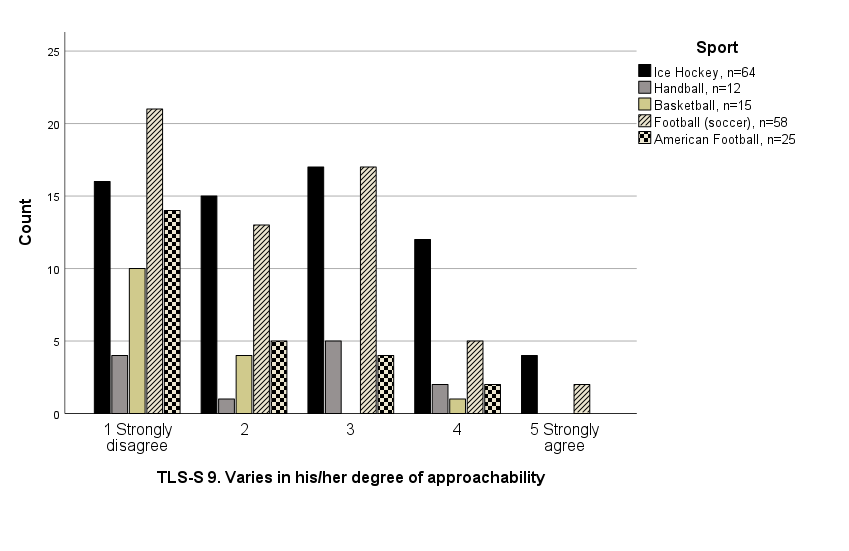
**

**
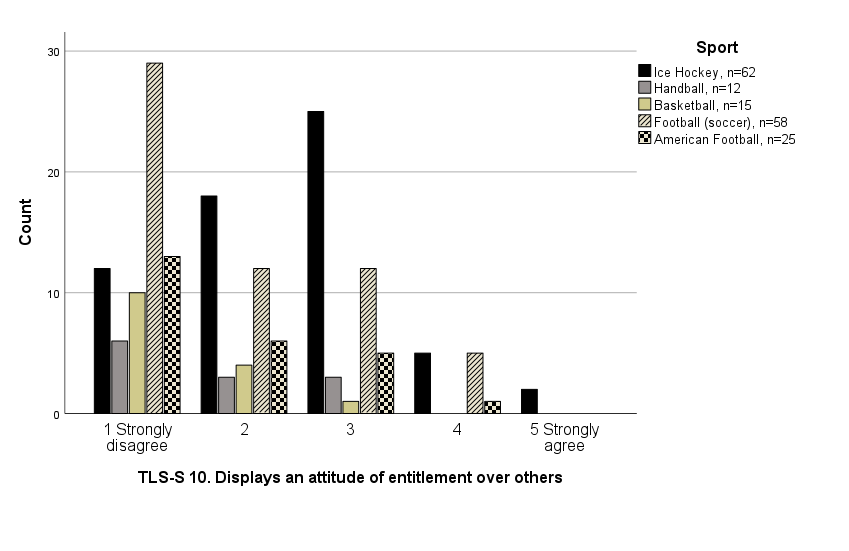
**

**
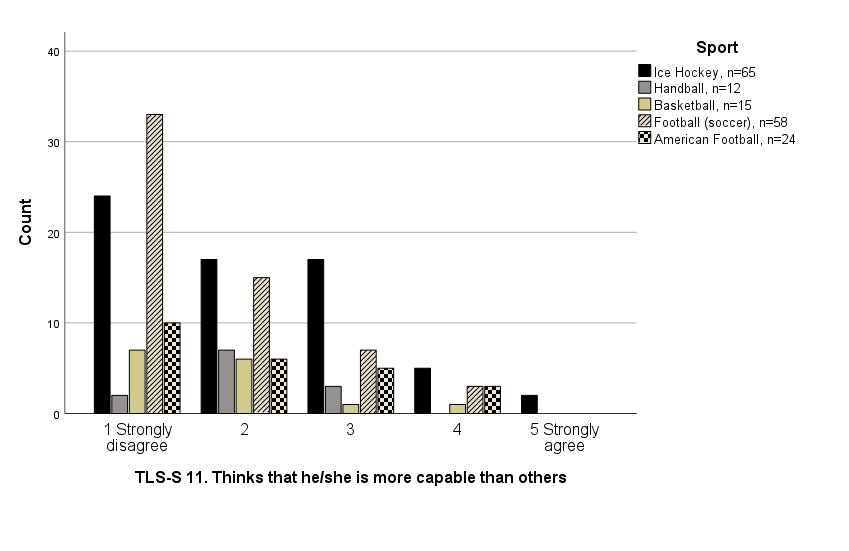
**

**
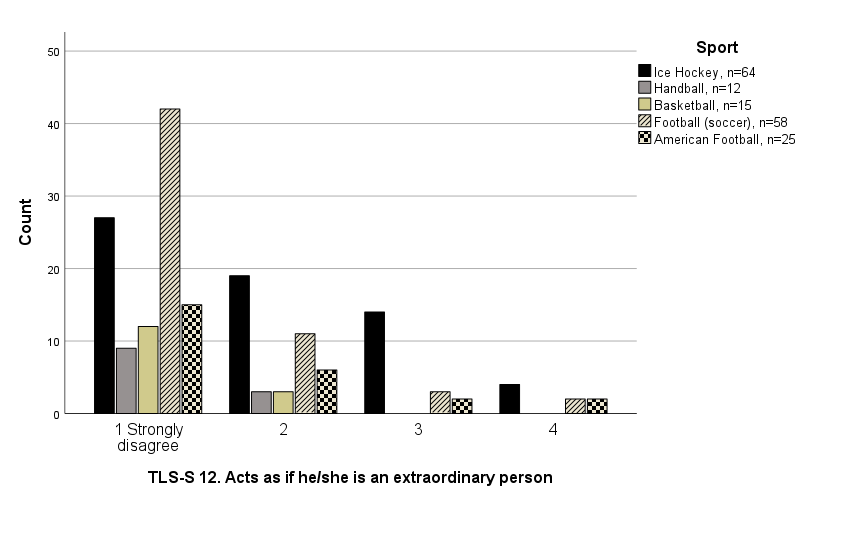
**

**
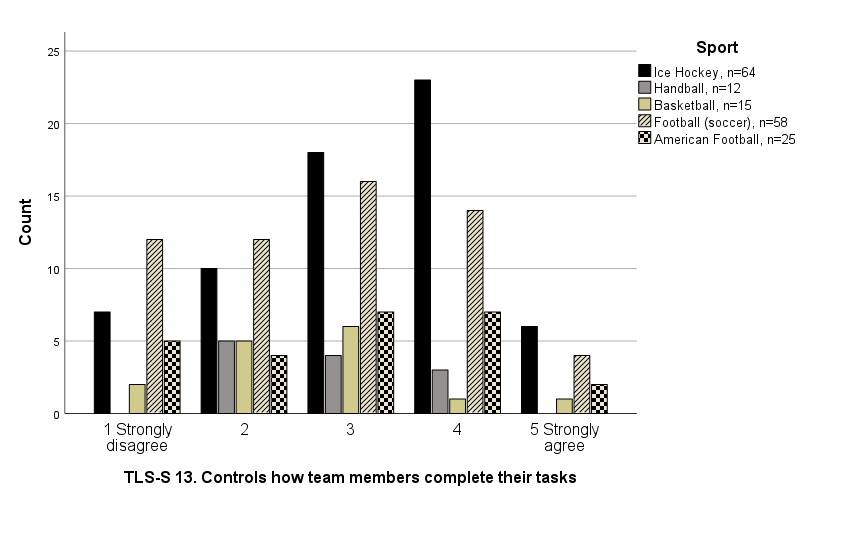
**

**
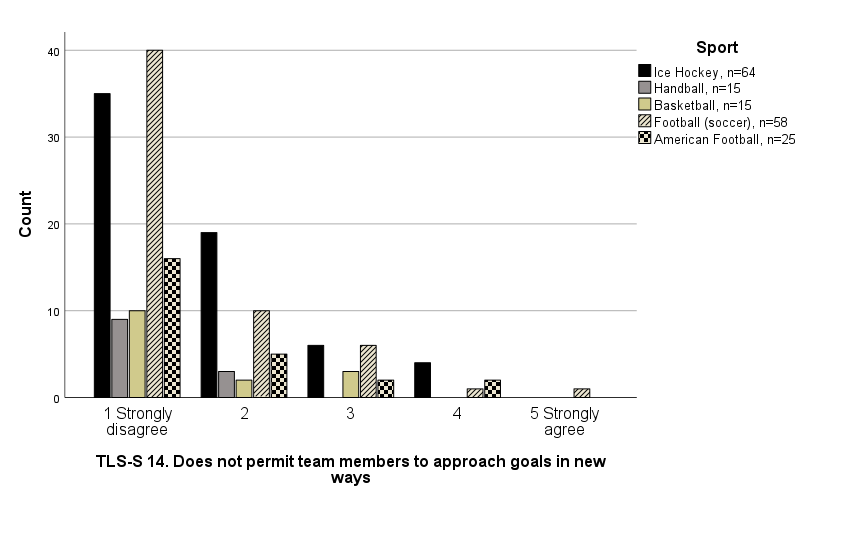
**

**
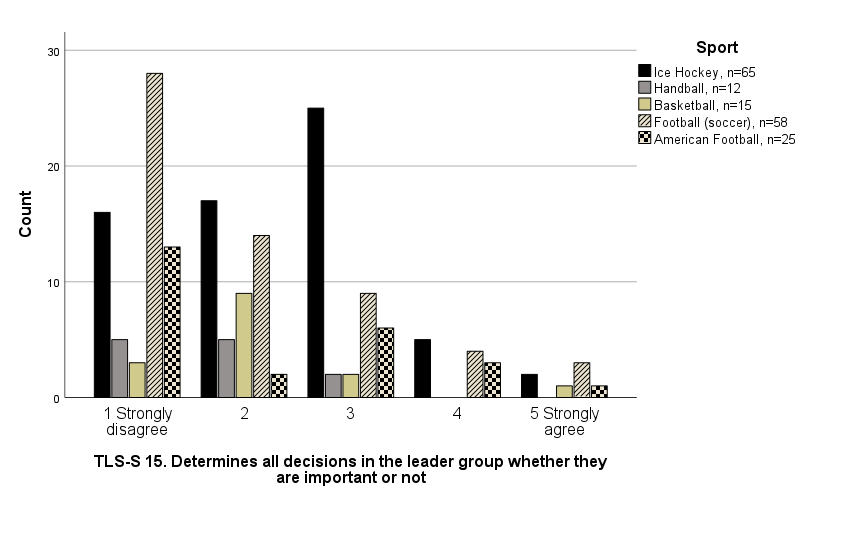
**

Supplement: S2 Appendix — Item-by-item analysis of the TLS‑S by gender and sports. (DOCX) [file pone.0343533.s002.docx]
